# Evaluation of Treatment Approaches Adopted by General Dentists for Early Childhood Caries

**DOI:** 10.7759/cureus.110210

**Published:** 2026-06-03

**Authors:** Pranitha Vadigireddy Gari, Anuchoudary Vattikuti, Akshat A Chokshi, Srinu Kashana, Mukesh Singla, Krishna Vallabhaneni

**Affiliations:** 1 Department of Public Health, College of Health Professions, The University of Southern Mississippi, Hattiesburg, USA; 2 Department of Clinical Informatics, Chiricahua Community Health Centers, Douglas, USA; 3 Department of Dentistry, Taunton Dental Village, Oshawa, CAN; 4 Department of Dentistry, Global Smiles Dental, Indianapolis, USA; 5 Department of Orthodontics, Mukesh's Dent-O-Care, Jagraon, IND; 6 Department of Pedodontics and Preventive Dentistry, KIMS Dental College, Amalapuram, IND

**Keywords:** atraumatic restorative treatment, dental caries, early childhood caries, pediatric dentistry, preventive dentistry

## Abstract

Introduction: Early childhood caries (ECC) remains a major challenge in pediatric oral healthcare because of its high prevalence and complex treatment needs. The present study aimed to compare the treatment options adopted by general dentists for the management of ECC and to evaluate the influence of dentist experience, child age, and caries severity on treatment selection.

Materials and methods: This cross-sectional, observational study was conducted among 150 general dentists between October 2021 and March 2022. Each dentist was observed during five consecutive consultations involving children aged two to five years diagnosed with ECC, resulting in 750 observed cases. Treatment modalities were categorized as fluoride varnish/remineralization therapy, atraumatic restorative treatment (ART), conventional restorations, preformed metal crowns, and extraction. Using a standardized observation checklist, data were recorded on dentist experience, child age, the dmft index (where d represents decayed primary teeth, m indicates missing teeth due to caries, f denotes filled primary teeth, and t stands for teeth), and parental presence. All analyses were performed using chi-square tests.

Results: Among the participating dentists, 112 (74.7%) practiced in private clinics, whereas parental presence during consultations was observed in 531 (70.8%) cases. Dentists with less than five years of experience more frequently selected fluoride varnish/remineralization (n = 74, 30.8%) and ART (n = 64, 26.7%), whereas dentists with more than 10 years of experience preferred preformed metal crowns and extractions (p < 0.001). Younger children aged two to three years predominantly received preventive treatments, whereas older children aged four to five years more commonly underwent conventional restorations and preformed metal crowns (p < 0.001). Increasing dmft severity was significantly associated with invasive treatment approaches, including extractions in the high dmft group (p < 0.001).

Conclusion: Treatment selection for ECC varied significantly according to dentist experience, age, and severity. Preventive and minimally invasive approaches are commonly adopted in younger children and in less severe cases, whereas invasive procedures increase with the severity of the disease. These findings highlight the importance of evidence-based minimally invasive strategies in pediatric dental practice.

## Introduction

Early childhood caries (ECC) is one of the most prevalent chronic diseases affecting preschool children worldwide and remains a significant public health concern because of its impact on pain, nutrition, growth, speech, and quality of life [1,2]. ECC is characterized by the presence of one or more decayed, missing, or filled tooth surfaces in any primary tooth in children younger than six years of age [3]. Despite advances in preventive dentistry and parental awareness programs, ECC continues to show a high prevalence, particularly in developing countries where socioeconomic disparities, inadequate oral hygiene practices, frequent sugar consumption, and limited access to pediatric dental care contribute to the disease burden [1,3]. ECC management is often challenging because treatment decisions depend on multiple factors, including the child’s age and behavior, caries severity, parental cooperation, available clinical resources, and the dentist’s experience and preferences [3].

General dentists play a major role in the early diagnosis and management of ECC, particularly in areas with limited access to pediatric dental specialists. Various treatment modalities are currently available for ECC management, ranging from noninvasive preventive approaches, such as fluoride varnish and remineralization therapy, to minimally invasive techniques, such as atraumatic restorative treatment (ART), conventional restorations, preformed metal crowns, and extractions in advanced cases [4-6]. The selection of an appropriate treatment modality is essential because it directly influences treatment success, patient comfort, long-term oral health outcomes, and preservation of the primary dentition. However, variations in clinical decision-making among general dentists are frequently observed, owing to differences in clinical training, years of experience, and perception of disease severity. Understanding these treatment patterns is important for evaluating adherence to evidence-based pediatric dental practices and identifying areas requiring additional training or clinical guidelines.

Therefore, the present study was conducted to compare the treatment options adopted by general dentists for the management of ECC in children aged two to five years. Therefore, the primary objective of the present study was to compare the treatment options adopted by general dentists for the management of ECC among children aged two to five years. The secondary objectives were to evaluate the association between treatment selection and dentist experience level, child age, and caries severity.

## Materials and methods

Study design and setting

This cross-sectional observational study was conducted at KIMS Dental College, Amalapuram, India, over a period of six months from October 2021 to March 2022. Ethical approval for the study was obtained from the institutional ethical committee (approval no: EC//KIMS/0508/2021/028) prior to commencement of data collection. Written informed consent was obtained from all participating dentists as well as from the parents or guardians of the children included in the study. Confidentiality and anonymity of both patient and dentist information were maintained throughout the study period, and all collected data were used solely for research purposes.

Study participants and sampling

In total, 150 general dentists were recruited using stratified random sampling from a regional dental registry. Dentists were eligible to participate if they had a minimum of one year of clinical experience, were actively involved in treating pediatric patients, and practiced in a non-specialized general dental setting. Dentists working exclusively in pediatric specialty clinics or tertiary care hospital settings were excluded from the study.

Sample size estimation was performed using G*Power version 3.1.9.7 (Heinrich Heine University Düsseldorf, Düsseldorf, Germany). Based on an expected moderate effect size (0.30) for chi-square goodness-of-fit analysis, with 80% statistical power and a significance level of 5%, the minimum required sample size was calculated as 138 participants [7]. After accounting for an anticipated 10% non-response or incomplete observation rate, the final sample size was rounded to 150 dentists.

Observation procedures

Each participating dentist was observed during routine clinical consultations involving five consecutive pediatric patients diagnosed with ECC. The observations were conducted using a standardized observation checklist by trained research assistants who did not interfere with the clinical procedures or treatment planning (see Appendices). Prior to data collection, all observers underwent calibration sessions to standardize recording procedures and treatment categorization. Standardized criteria based on routine clinical examination and the decayed, missing, and filled teeth (dmft) index assessment were used for ECC diagnosis and caries severity classification. The primary treatment modality selected by the dentist for each child is documented. Treatment options were categorized into five groups: fluoride varnish or remineralization therapy, ART, conventional restorations using composite resin or glass ionomer cement materials, preformed metal crowns, and extraction under local anesthesia. The observational approach enabled the assessment of real-world treatment preferences adopted by general dentists during routine pediatric dental care.

Data collection and study variables

Data collection was performed using structured paper-based recording forms, followed by double entry into an electronic database to minimize transcription errors. The primary outcome variable was the frequency of treatment options selected for ECC management. Secondary variables included dentist-related factors, such as years of clinical experience and practice setting, as well as child-related variables, including age; caries severity assessed using the dmft index; and parental presence during clinical decision-making [8]. Cases were categorized into low (dmft score 1-2), moderate (dmft score 3-4), and high (dmft score ≥5) groups for comparative analysis [8].

Statistical analysis

Data analysis was performed using IBM SPSS Statistics for Windows, Version 25 (Released 2017; IBM Corp., Armonk, New York, United States). Descriptive statistics, including frequencies, percentages, means, standard deviations, medians, and interquartile ranges, were used to summarize the demographic characteristics and treatment choices. Associations between treatment options and dentist experience level, child age group, and caries severity were evaluated using Pearson’s chi-square test or Fisher’s exact test, wherever appropriate. Inter-observer reliability for the standardized observation checklist was assessed using Cohen’s kappa statistics. A kappa value of 0.91 was obtained, indicating excellent agreement between observers. Statistical significance was set at a p-value less than 0.05.

## Results

A total of 150 general dentists participated in this cross-sectional observational study. Among them, 82 (54.7%) were males, and 68 (45.3%) were females. The mean age was 34.6 ± 7.2 years, with a median of 33 years (IQR 29-39). Clinical experience averaged 7.4 ± 4.2 years; 48 dentists (32.0%) had less than five years of experience, 57 (38.0%) had 5-10 years, and 45 (30.0%) had more than 10 years. Regarding practice setting, 112 (74.7%) worked in private clinics and 38 (25.3%) in government clinics. During 750 observed child consultations (five per dentist), parental presence was recorded in 531 cases (70.8%), while 219 cases (29.2%) had no parent present, as shown in Table 1. These demographic characteristics represent a diverse sample of general dentists with varying experience levels and practice backgrounds.

**Table 1:** Demographic and professional characteristics of participating dentists (n = 150). Data are presented as n (%) or mean ± standard deviation (SD) unless otherwise specified; IQR: interquartile range; cases: child consultations observed (150 dentists × 5 cases per dentist = 750 observed cases); parental presence data are based on total observed consultations (N = 750).

Variable	Category	Value
Sex, n (%)	Male	82 (54.7)
	Female	68 (45.3)
Age (years)	Mean ± SD	34.6 ± 7.2
	Median (IQR)	33 (29-39)
	Range	24-58
Clinical experience (years)	Mean ± SD	7.4 ± 4.2
	Median (IQR)	7 (4-11)
Clinical experience, n (%)	<5 years	48 (32.0)
	5-10 years	57 (38.0)
	>10 years	45 (30.0)
Practice setting, n (%)	Private clinic	112 (74.7)
	Government clinic	38 (25.3)
Parental presence during consultation (for 750 cases), n (%)	Yes	531 (70.8)
	No	219 (29.2)

**Figure 1:**
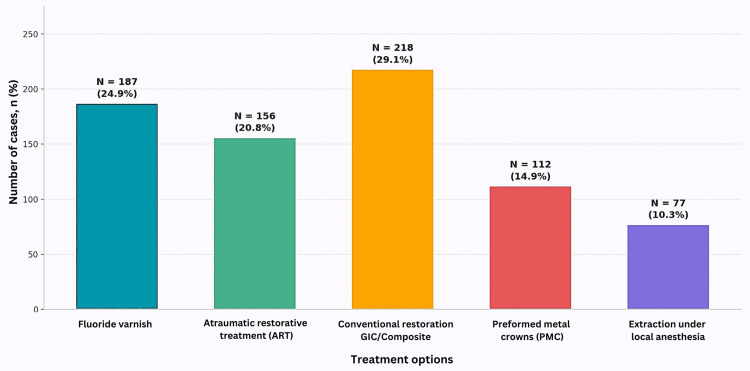
Overall frequency distribution of treatment options for early childhood caries (ECC) observed across all cases. GIC: glass ionomer cement; PMC: preformed metal crown

The overall frequency distribution of treatment options adopted for ECC management showed that conservative and minimally invasive treatment approaches were more commonly selected than invasive procedures, with conventional restorations being the most frequently used treatment modality, whereas extraction is the least preferred option (Figure 1).

Table 2 shows a statistically significant association between dentist experience and treatment selection (p = 0.001). Dentists with less than five years of experience preferred fluoride varnish/remineralization and ART, whereas dentists with more than 10 years of experience more commonly selected conventional restorations, followed by preformed metal crowns.

**Table 2:** Distribution of treatment options according to dentist experience level. Data are presented as n (%); Pearson chi-square test used for comparison; *p < 0.05: statistically significant; experience groups included <5 years (240 cases), 5-10 years (285 cases), and >10 years (225 cases); ART: atraumatic restorative treatment

Treatment option	<5 years (n = 240), n (%)	5-10 years (n = 285), n (%)	>10 years (n = 225), n (%)	Chi stats	p-value
Fluoride varnish/remineralization	74 (30.8)	72 (25.3)	41 (18.2)	126.7	0.001*
ART	64 (26.7)	59 (20.7)	33 (14.7)		
Conventional restorations	64 (26.7)	84 (29.5)	70 (31.1)		
Preformed metal crowns	24 (10.0)	44 (15.4)	44 (19.6)		
Extraction	14 (5.8)	26 (9.1)	37 (16.4)		

Table 3 presents the distribution of treatment options according to age group. Younger children aged two to three years more frequently received fluoride varnish/remineralization and ART, whereas older children aged four to five years more commonly underwent conventional restorations and fluoride varnish followed by ART. A statistically significant difference in treatment selection between the age groups was observed (p < 0.001).

**Table 3:** Distribution of treatment options according to child age group. Data are presented as n (%); Pearson chi-square test used for comparison between age groups; df: degrees of freedom; *p < 0.05: statistically significant; ART = atraumatic restorative treatment.

Treatment option	2-3 years (n = 350), n (%)	4-5 years (n = 400), n (%)	χ^2^ (df)	p-value
Fluoride varnish/remineralization	112 (32.0)	75 (18.8)	29.23	<0.001*
ART	84 (24.0)	72 (18.0)		
Conventional restorations	82 (23.4)	136 (34.0)		
Preformed metal crowns	44 (12.6)	68 (17.0)		
Extraction	28 (8.0)	49 (12.3)		

Table 4 shows the association between caries severity and treatment choice. Children with low dmft scores predominantly received preventive treatment modalities such as fluoride varnish/remineralization and ART, whereas more than 50% of children with high dmft scores underwent invasive procedures including preformed metal crowns and extractions. The association between dmft severity and treatment choice was significant (p = 0.001).

**Table 4:** Distribution of treatment options according to caries severity (dmft score). Data are presented as n (%); Pearson chi-square test used for comparison; *p < 0.05: statistically significant; low dmft: dmft score 1-2; moderate dmft: dmft score 3-4; high dmft: dmft score ≥5; dmft: decayed, missing, and filled teeth; ART: atraumatic restorative treatment

Treatment option	Low dmft (n = 250), n (%)	Moderate dmft (n = 310), n (%)	High dmft (n = 190), n (%)	χ^2^ (df)	p-value
Fluoride varnish/remineralization	98 (39.2)	67 (21.6)	22 (11.6)	151.13	0.001*
ART	72 (28.8)	68 (21.9)	16 (8.4)		
Conventional restorations	57 (22.8)	107 (34.5)	54 (28.4)		
Preformed metal crowns	15 (6.0)	47 (15.2)	50 (26.3)		
Extraction	8 (3.2)	21 (6.8)	48 (25.3)		

For the standardized observation checklist, interobserver agreement was assessed across 750 observed consultations, with concordance achieved in 715 instances (95.3%). The resulting Cohen’s kappa value was 0.91, reflecting an almost perfect level of agreement between the observers.

## Discussion

ECC continues to represent a major pediatric oral health challenge globally, particularly in developing countries where delayed diagnosis and limited access to specialized pediatric dental care often result in advanced disease presentation [3]. The present study evaluated the treatment options adopted by general dentists for managing ECC and demonstrated significant variations in treatment selection based on dentist experience, age, and caries severity. These findings provide valuable insights into contemporary clinical decision-making patterns among general dental practitioners involved in pediatric oral healthcare.

The present study showed that conservative and minimally invasive approaches, including fluoride varnish/remineralization therapy and ART, are frequently selected for ECC management [4-6]. This trend may reflect the increasing awareness among general dentists regarding preventive pediatric dentistry and minimally invasive treatment concepts aimed at preserving tooth structure and improving child cooperation. These findings are consistent with those of the study by Takriti et al. [7], who reported that general dentists increasingly emphasize preventive interventions and noninvasive management strategies for ECC, particularly in early lesions and younger children. Preventive approaches are often preferred because they reduce treatment-related anxiety, preserve primary dentition, and can be delivered effectively in routine clinical settings.

A significant association was observed between dentist experience and treatment choices. Dentists with less than five years of experience more frequently selected preventive modalities and ART, whereas more experienced practitioners preferred preformed metal crowns and extractions [9,10]. This pattern may be attributed to differences in educational exposure, clinical confidence, and interpretation of disease progression. Younger practitioners are often trained under modern minimally invasive dentistry protocols and may demonstrate greater adherence to the current preventive guidelines. Conversely, more experienced dentists may rely on traditional restorative approaches and prefer definitive treatment options for long-term durability and reduced retreatment risks. Similar observations were reported by Tinanoff and Reisine [11], who noted that treatment philosophies among dental practitioners often vary according to clinical training and years of practice experience.

The present study also demonstrated significant age-related differences in treatment selection. Younger children aged two to three years predominantly received fluoride varnish and ART, while older children aged four to five years more commonly underwent conventional restorations, preformed metal crowns, and extractions [12]. This finding likely reflects the progressive nature of ECC, where lesions in younger children are frequently diagnosed at earlier stages, and can therefore be managed conservatively. In older children, delayed presentation and prolonged disease activity often result in greater cavitation and structural tooth destruction, necessitating invasive restorative procedures. Similar age-related treatment trends have been reported in previous epidemiological studies evaluating pediatric restorative care patterns [13].

Caries severity assessed using dmft scores showed a strong association with treatment decisions. Children with low dmft scores mainly received preventive and minimally invasive interventions, whereas those with high dmft scores more frequently required invasive treatments such as preformed metal crowns and extractions. This progression from preventive care to definitive restorative management is clinically expected because increasing caries severity is associated with greater enamel and dentinal destruction, pulpal proximity, pain, and compromised tooth prognosis. These findings are in agreement with the recommendations of the American Academy of Pediatric Dentistry, which advocates risk-based and lesion-specific treatment planning for ECC management [14]. Previous studies by Frencken et al. [15] and Gao et al. [16] similarly reported that minimally invasive approaches are the most effective in low-to-moderate caries risk groups, whereas advanced disease frequently requires extensive restorative intervention.

The high interobserver agreement obtained in the present study indicates the strong reliability of the standardized observation checklist and supports the methodological validity of the collected data. Consistency between observers minimizes measurement bias and strengthens the accuracy of treatment categorization during clinical observations.

Our findings have several important clinical implications. The observed preference for minimally invasive strategies among younger dentists suggests an encouraging shift toward preventive pediatric dental care. However, variability in treatment decisions among practitioners highlights the need for standardized evidence-based clinical guidelines and continuing dental education programs that focus on ECC management. Strengthening training in minimally invasive dentistry, behavioral management, and early caries detection may further improve treatment consistency and patient outcomes. Additionally, public health initiatives promoting early dental visits and parental awareness may help reduce ECC progression and the need for invasive procedures in preschool children.

Despite its strengths, this study has several limitations. The cross-sectional observational design limits the ability to evaluate long-term treatment outcomes, patient satisfaction, and causal relationships following different treatment modalities. The study was restricted to general dental clinics within a single geographic region, which may have affected the generalizability of the findings to other populations and healthcare systems. In addition, practice location variables such as urban and rural settings were not assessed, and therefore their potential influence on treatment decision-making could not be evaluated. Furthermore, factors such as socioeconomic status, parental education, child behavior, and access to dental insurance were not assessed, although these variables may significantly influence treatment selection. Although standardized observation checklists and high inter-observer agreement were maintained, the possibility of observer-related bias cannot be completely excluded. Repeated observations from the same dentists may also have introduced clustering effects that were not specifically adjusted for statistically. Future multicenter longitudinal studies incorporating broader demographic, behavioral, and geographic variables are recommended to better understand the determinants of ECC treatment selection and clinical outcomes.

## Conclusions

The present study demonstrated significant variations in the treatment approaches adopted by general dentists for the management of ECC. Preventive and minimally invasive procedures were more commonly selected for younger children and patients with lower caries severity, whereas invasive treatments were more frequently observed in older children, cases with higher dmft scores, and among dentists with greater clinical experience. These findings highlight important associations in clinical decision-making patterns and emphasize the importance of early diagnosis, evidence-based treatment planning, and continuous professional training to promote consistent, minimally invasive, and child-centered management strategies for ECC.

## Appendices

**Table 5:** Standard observer checklist.

Part 1	
Observer Name:	
Date of Observation:	
Clinic ID:	
Part 2	
Observation Parameter	Options (choose any one)
Dentist ID (or code)	Mention allotted dentist ID.
Dentist’s clinical experience	□ <5 years □ 5-10 years □ >10 years
Practice setting	□ Private clinic □ Government clinic
Child’s age	□ 2 years □ 3 years □ 4 years □ 5 years
Child’s dmft score	Mention calculated dmft score.
Caries severity category	□ Low (1-2) □ Moderate (3-4) □ High (≥5)
Parental presence during consultation	□ Yes □ No
Primary treatment modality selected	□ Fluoride varnish/remineralization □ ART □ Conventional restoration (GIC/composite) □ Preformed metal crown □ Extraction
Tooth/teeth involved	Mention tooth numbers.
Observer agreement note	□ Observer 1 □ Observer 2
